# Rapid single step subcloning procedure by combined action of type II and type IIs endonucleases with ligase

**DOI:** 10.1186/1754-1611-1-7

**Published:** 2007-11-26

**Authors:** Tobias Fromme, Martin Klingenspor

**Affiliations:** 1 Department of Animal Physiology, Faculty of Biology, Philipps-University, D-35043 Marburg, Germany

## Abstract

**Background:**

The subcloning of a DNA fragment from an entry vector into a destination vector is a routinely performed task in molecular biology labs.

**Results:**

We here present a novel benchtop procedure to achieve rapid recombination into any destination vector of choice with the sole requirement of an endonuclease recognition site. The method relies on a specifically designed entry vector and the combined action of type II and type IIs endonucleases with ligase. The formulation leads to accumulation of a single stable cloning product representing the desired insert carrying destination vector.

**Conclusion:**

The described method provides a fast single step procedure for routine subcloning from an entry vector into a series of destination vectors with the same restriction enzyme recognition site.

## Background

One of the most routinely performed tasks in molecular biology labs around the world undoubtedly is the subcloning of a given DNA fragment from one plasmid vector into a different one. The reasons to do so are as numerous and diverse as are the applied methods. We here describe a further such technique involving the orchestrated action of a typeII and a typeIIs endonuclease ("outside cutter") with ligase. This procedure achieves the speed of recombinase based methods without the need for unusual recognition elements in the target vector.

## Method

**Figure 1:**
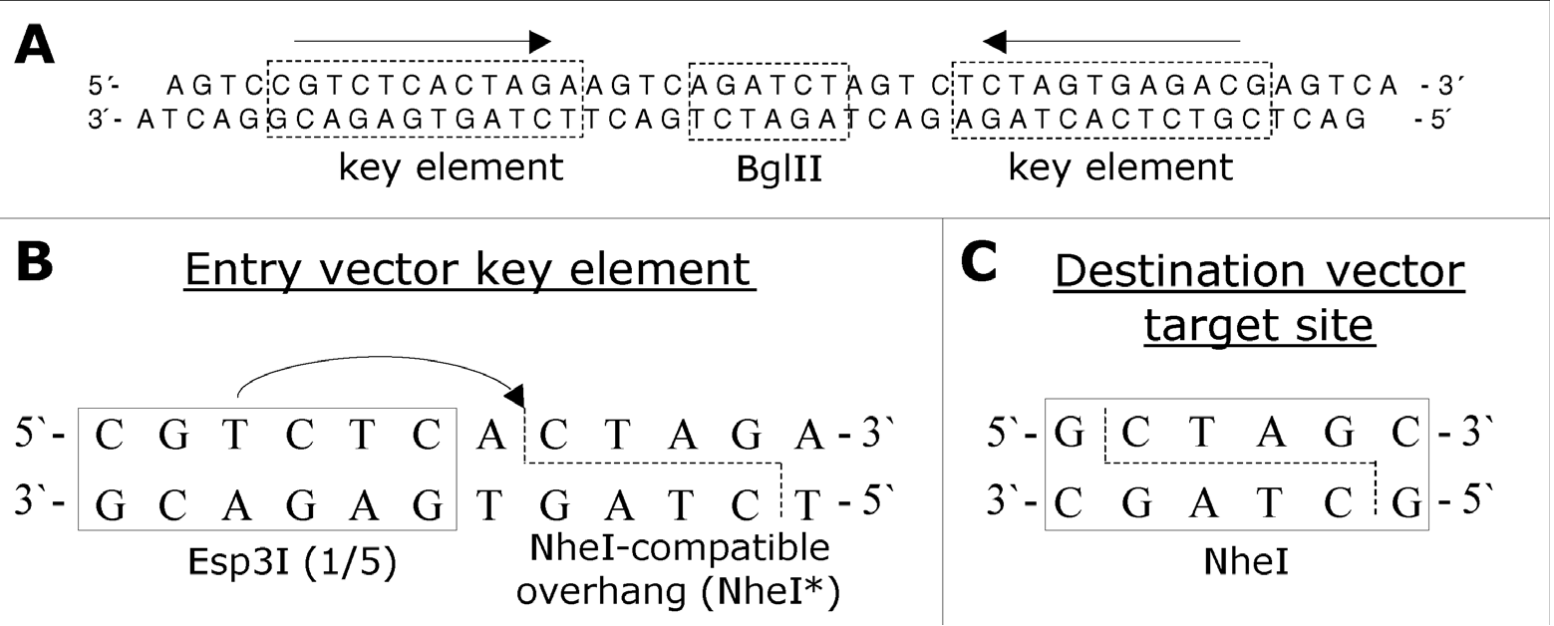
**Sequence elements facilitating the subcloning procedure**. (A) A double stranded oligonucleotide was cloned into pGEM-T easy to provide two key elements flanking a BglII site. We inserted a 1166 bp PCR amplified fragment into this restriction site. In future applications this step may as well be achieved by TA cloning or other methods. (B) A key element consists of a Esp3I recognition site and a restriction site generating a NheI compatible overhang, that is *not *recognized by NheI. (C) We used NheI as target recognition site in the destination vector.

In an example experiment we subcloned a 1166 bp long DNA fragment* from an entry vector (modified pGEM-T easy, Promega) into a NheI site of a destination vector (pEGFP-N1, Clontech). The method relies on a specifically engineered entry vector which comprises two key elements flanking the insert to be subcloned (Figure 1A). To test our method we accordingly modified the plasmid pGEM-T easy and included a BglII site to be able to insert a DNA fragment between these elements. A blunt end cutting enzyme to Taq-generate T overhangs for TA cloning of PCR products or any other means to insert a fragment of choice are of course feasible as well. Both of the two identical key elements comprise a recognition sequence for a type IIs restriction endonuclease – Esp3I in our case – and a restriction site (Figure 1B). The latter one is specifically designed to yield a NheI-compatible single stranded overhang upon digestion with Esp3I. It is essential to design the key element restriction site in a way that the bases directly adjacent to the single stranded overhang are different from those in a NheI recognition site (compare Figure 1B and 1C).

**Figure 2:**
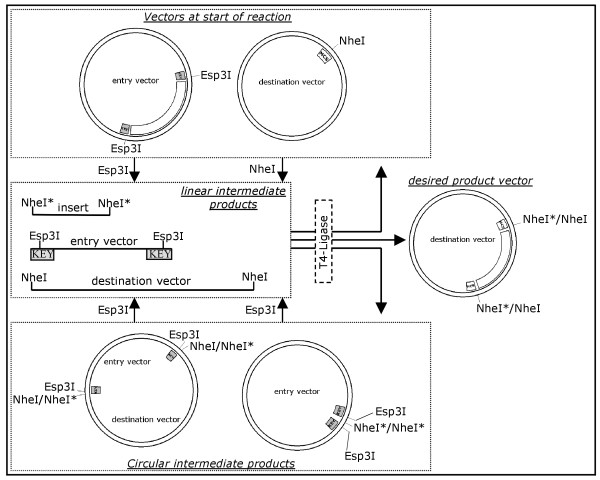
**Schematic overview of the subcloning procedure**. The upper box contains the two vectors the reaction starts with, i.e. the entry vector with its two key elements flanking an insert and the destination vector with its NheI recognition site. By the enzymatic action (arrows) of Esp3I and NheI these vectors are linearized to form linear intermediate products as shown in the central box. These intermediates are subject to T4 ligase activity and can be ligated to yield a range of products: the initial vectors (upper box), circular intermediate products (lower box) and the desired product vector. Note that all circular intermediate products are again substrate for Esp3I and thus again linearized. There is a sole stable product in this reaction system, which is the desired product vector (circular products only shown if carrying at least one resistance marker). Shaded boxes termed KEY: key element as shown in Fig. 1.

We combined entry vector, destination vector, Esp3I (Fermentas), NheI (Fermentas) and T4 ligase (Promega) in a buffer allowing all three enzyme actions (**) and incubated at room temperature for 1 hour (Figure 2). We transformed 2 μl of the resulting solution into DH5α chemically competent E. coli (Invitrogen) and plated on two plates either containing kanamycin or ampicillin.

## Results & Discussion

In three independent tests we found between 40 and 73 colonies on the kanamycin plate (indicating pEGFP-N1) and no colonies at all (in two tests) or 10 colonies (in one test) on the ampicillin plate (harbouring pGEM-Teasy). Of 10 colonies per kanamycin plate tested for the presence of the subcloned insert in pEGFP-N1 by restriction analysis all proved to be the desired product. Noteworthy the two different resistance markers on entry and destination vector are not necessary for the procedure to function but were used for the sole purpose of easy discrimination between products in our tests. Following this proof of principle the procedure can certainly be further refined.

The entry vector once constructed can only serve to subclone into one selected restriction site. Further limitations of this technique are the inapplicability for directional cloning and the dependence on absence of the utilized endonuclease recognition sites on both vector backbone and insert. We feel, however, that the speed of this single-step benchtop procedure makes up for this when routinely cloning PCR products into a variety of destination vectors all containing the same restriction site. Our group for instance is interested in cloning certain promoter elements upstream of several different reporter genes and conversely, cloning a given open reading frame behind a series of different promoters. An even broader application can be envisaged in the field of engineering improved enzymes by gene shuffling approaches or, more generally, as one instrument in the molecular toolbox enabling the construction processes that essentially constitute synthetic biology.

## Footnotes

* Genbank AY523564 from BglII site at base position 2325 to BglII site at 3491.

** Y+/tango-buffer (Fermentas; 33 mM Tris acetate, 10 mM Mg-acetate, 66 mM K-acetate, 0.1 g/L BSA) supplemented with 5% (w/v) PEG, 10 mM DTT and 1 mM rATP in a final volume of 10 μL. Enzyme amounts: 7.5 units Esp3I, 10 units NheI, 3 weiss units T4 ligase.

## Competing interests

The author(s) declare that they have no competing interests.

## Authors' contributions

TF devised the described procedure, conducted all experiments and drafted the manuscript. MK provided the tangible base and technical expertise in refining this method and assisted in drafting the manuscript. Both authors read and approved the final version of this manuscript.

